# Overcoming barriers to recruiting ethnic minorities to mental health research: a typology of recruitment strategies

**DOI:** 10.1186/s12888-015-0484-z

**Published:** 2015-05-02

**Authors:** Waquas Waheed, Adwoa Hughes-Morley, Adrine Woodham, Gill Allen, Peter Bower

**Affiliations:** 1 Centre for Primary Care, Institute of Population Health, University of Manchester, Williamson Building, Oxford Road, Manchester, M13 9PL UK; 2 Lancashire Care NHS Foundation Trust, Preston, UK; 3 Department of Psychology, University of Bolton, Bolton, UK; 4 MRC North West Hub for Trials Methodology Research, Manchester Academic Health Science Centre, University of Manchester, Manchester, UK

## Abstract

**Background:**

The ethnic minority population in developed countries is increasing over time. These groups are at higher risk of mental illness and demonstrate lower participation in research. Published evidence suggests that multiple factors like stigma, lack of trust, differences in explanatory models, logistical issues and lack of culturally aware researchers act as barriers to ethnic minority recruitment into mental health research. To reduce inequalities in participation, there is a need to devise innovative and culturally sensitive recruitment strategies. It is important that researchers share their experience of employing these strategies so that ethnic minority participation can be facilitated.

**Methods:**

We previously published a systematic review of barriers to recruiting ethnic minority participants into mental health research. The nine papers included in our prior review formed the basis for developing a typology of barriers to recruiting ethnic minorities into mental health research. This typology identified 33 barriers, described under five themes. We further extracted data on the strategies used to overcome these recruitment barriers, as described in the included studies.

**Results:**

The strategies employed by the authors could be matched to all but two barriers (psychopathology/substance misuse and limited resource availability). There was evidence that multiple strategies were employed, and that these depended upon the population, clinical set-up and resources available.

**Conclusions:**

This typology of strategies to overcome barriers to recruiting ethnic minorities provides guidance on achieving higher rates of recruitment. It is important that researchers plan to deploy these strategies well in advance of initiating recruitment. Whilst adopting these strategies, the authors have not been able to quantify the positive impact of these strategies on recruitment. The typology should encourage researchers to employ these strategies in future research, refine them further and quantitatively evaluate their impact.

## Background

Due to continuous migration, developed countries are becoming more diverse in their ethnic make-up, and the population of ethnic minority groups is increasing over time [1]. Individuals from ethnic minority groups are known to be at higher risk of mental illness, to underutilise health services and to have lower participation in health research [2-4]. A systematic review of all published randomised controlled trials in panic disorder showed that only 44.7% reported ethnicity data for their included sample [5]. Only 24% of US and Canadian studies have included Latino participants [6], demonstrating a substantial underrepresentation of Latinos in clinical studies for OCD [7]. Difficulties with recruiting research participants from ethnic minority groups is not solely restricted to mental health research; such problems have been encountered in range of other areas including research in nursing [8], public health [9] and cardiovascular disease [10]. This low rate of participation among ethnic minorities reduces the generalisability of mental health research findings [11] and impacts on the development of effective services and interventions [9], leading to further widening of health inequalities [12].

African-American adults have voiced their concerns about mistrust as the dominating barrier to research participation [13]. However a review of research enrolment decisions of over 70,000 participants found ethnic minorities to be equally willing to participate in research [14]. There is an immediate need to work towards developing appropriate strategies to improve participation among ethnic minorities in research [12].

We published the only systematic review summarising barriers to the recruitment of ethnic minorities into mental health research [15]. This review listed multiple barriers to recruitment and grouped the barriers into overarching themes, which we summarise in Table 1.

**Table 1:** **Summary of Barriers to recruiting ethnic minorities to mental health research** ^**10**^

**Participant related barriers**	**Family/ community related**
1. Explanatory models of illness	18. Husbands’ influence
2. Help-seeking/- ive attitude to psychotherapy	19. Family perspectives
3. Language spoken	20. Stigma for family
4. Religious beliefs and commitments	**Health services related**
5. Trust	21. Utilisation of mental health service
6. Stigma	22. Language of professional/intervention
7. Gender	23. Communication and cultural awareness between staff and participants
8. Psychopathology/Substance Misuse	
9. Fear of being reported to immigration	24. Staff personal attributes
**Practical Issues**	**Research process related**
10. Lack of childcare	25. Limited willingness and enthusiasm
11. Transport provision	26. Understanding the need for ethnic participation
12. Financial constrains	
13. Culturally appropriate incentive	27. Paucity of resources available
14. Medical insurance	28. Appropriateness of assessment tools
15. Lack of time	29. Non availability of translated materials
16. Location of interview	30. Lack of culturally competent staff
17. Employment status	31. Lack of culturally matched staff
	32. Under representation at recruitment Sites
	33. Understanding of consent process

As a next step, we wanted to understand what recruitment strategies were recommended for overcoming barriers to recruiting ethnic minorities into mental health research. We extracted data from all included studies in our review [15] with an aim to: (a) create a typology of recommended strategies to overcome barriers to research participation among ethnic minorities; and (b) match these recommended strategies to recruitment barriers. This will create a resource for researchers to assist with planning how to overcome recruitment barriers and increase confidence to enable the inclusion of more participants from ethnic minority groups.

## Method

We revisited the nine [16-24] papers included in our published systematic review describing the recruitment of ethnic minorities into clinical studies (including trials and non-trials) in mental health. In the review, we combined search terms from three domains in Medline, Embase, PsycINFO and Cinahl: (1) participation or recruitment or retention; (2) ethnic minorities (exploded) or culture; (3) research or trial. Key words within the three domains were combined using “OR” and “AND”. We included English-language articles only. We did not aim to focus this review on any particular ethnic group or country of residence. Therefore no specific search terms for ethnic minorities or country were used. For details of the systematic review methodology please see the published paper [15].

Data describing every strategy used to overcome recruitment barriers were extracted from each paper to create a typology of recommended strategies. Two authors (WW and AW) then matched these recruitment strategies to 33 recruitment barriers grouped into 5 broad categories that had been previously tabulated in our systematic review.

The matching of a recruitment strategy to a barrier is not mutually exclusive and any one strategy may be effective across several barriers, making some repetition within the review unavoidable.

## Results

In our published systematic review the initial search identified 10,089 papers, of which 9 were included. All nine included papers originated from the United States. A brief description and the barriers described in the nine papers are provided in Tables 2 and 3 [15].

**Table 2:** **Description of included papers**

Title	Setting	Ethnic group	Illness
Miranda et al. 1996 [16]	San Francisco, USA, Hospital	Latino	Depression
Thompson et al. 1996 [17]	Detroit, USA, Two hospitals	African American	Schizophrenia or mood disorder
Le et al. 2008 [18]	Mexico City, Mexico: Hospital, community health care centre	Latino	Postpartum depression
	Washington DC, USA: Hospital, community health care centre		
Arean et al. 2003 [19]	San Francisco Bay, USA, Community clinic	African American	Depression or anxiety
Meinert et al. 2003 [20]	Cleveland, USA, Community	African American	Depression
Gallagher-Thompson et al. 2004 [21]	San Francisco, USA, Community	Latino	Dementia
Chen et al. 2005 [22]	New York City, USA, Neighbourhood health centre	Asian American	Depression
Aliyu et al. 2006 [23]	South Eastern USA, Aacademic medical centres	African American	Schizophrenia & Schizoaffective disorder
Loue & Sajatovic. 2008 [24]	San Diego and North East Ohio, USA	Latino	Schizophrenia, bipolar disorder or major depression

**Table 3:** **Summary of proposed strategies**

		Miranda [ 16 ]	Thompson [ 17 ]	Le [ 18 ]	Arean [ 19 ]	Meinert [ 20 ]	Gallagher-Thompson [ 21 ]	Chen [ 22 ]	Aliyu [ 23 ]	Loue [ 24 ]
**Participant Related Barriers**	Explanatory models of illness							√		
	Help-seeking/-ve attitude to psychotherapy							√		
	Language spoken	√		√		√	√			√
	Religious beliefs and commitments				√			√		
	Lack of trust					√			√	√
	Stigma				√					√
	Gender issues	√								
	Psychopathology/Substance Misuse									
	Fear of being reported to immigration			√						
**Practical Issues**	Lack of Childcare	√						√		
	Transport Provision	√				√	√	√		
	Financial constrains		√			√	√	√		
	Culturally inappropriate incentives	√						√		√
	Medical insurance	√	√							
	Lack of time				√					√
	Location of interview				√					√
	Employment status							√		
**Family/Community Related**	Husbands’ Influence	√								
	Family perspectives	√						√		
	Stigma for family				√		√			
	Under utilisation of mental health service						√			
	Language of professional/intervention	√		√						
	Lack of communication and cultural awareness between staff and participants			√				√		
	Staff personal attributes	√		√			√		√	
**Research Process**	Limited willingness and enthusiasm	√		√			√	√		
	Understanding the need for ethnic participation							√		
	Paucity of resources available									
	Appropriateness of assessment tools		√				√			
	Non availability of translated materials	√						√		√
	Lack of culturally competent staff		√	√	√		√			√
	Lack of culturally matched staff		√		√					√
	Under representation at recruitment sites	√	√	√				√	√	√
	Understanding of consent process		√	√				√		

### Participant related barriers

#### Explanatory models of illness

##### Recruiting person to adopt a stepped approach to introducing the study [22]

i. Initially emphasize the link between stress and general health.
ii. Then highlight the importance of the topic for both the patient and the community.
iii. Once initial buy-in achieved, only then brings up issues of mental health.

##### Plan intensive outreach activities with the target community [22]

- Lectures on depression and anxiety, at local senior centres, home care agencies, and social service centres.
- Wrote public education articles on mental illness in the local newspapers.
- Distribute brochures on the prevention, diagnosis, and treatment of depression, anxiety, and alcohol disorders.
- Radio programs on research topics, which gave the advantage of wide and regular access.

##### Involve a culturally competent person

A culturally competent person who is viewed by the ethnic minority participant as an “insider” is best placed to explain the essentially Western concepts of mental disorders and treatment. That person can be a researcher, clinician or a community volunteer who can think and behave in a way that is sensitive to the needs of the participant [22]*.*

#### Help-seeking/negative attitude to psychotherapy

##### Educate the community about mental illness and use this opportunity to facilitate recruitment [22]

- Use case based descriptions in newspaper articles using culturally appropriate language. This is useful for relatively low literacy levels. Articles that solely announced new research programs attract the least attention.
- Post flyers and advertisements to increase visibility of the project.

#### Language spoken

##### Use bilingual and bicultural staff

This helps in better communication in participant’s preferred language [16,24] and provides greater sensitivity when selecting language and common expressions [20].

##### Appoint cultural consultants to improve the relevance of outreach materials [21]

*Ensure participants fully understand the intent and content of consent forms*

Certain terms can appear to be alarming to participants, so “*Investigación”* (investigation, instead of “study”) and “*el gobierno federal y otras personas en representación de la Universidad pueden revisar su información”* (“the federal government and other persons representing the University can review your information”) were rephrased accordingly [18].

##### Translating technical phrases

Numerous scientific phrases like “randomisation” are often difficult to translate or explain in another language. We tell people exactly what would happen to a patient, rather than dwelling on finding an appropriate translated name for the procedure. For example, patients are simply told *“…*afterwards, the patient will have an equal chance of going to either of the following two treatment models …” [22].

#### Religious beliefs

##### Collaborate with religious leaders

The study team invited researchers, mental health consumers and pastors of Chinese churches to form a project advisory council [22].

##### Be aware of religious denominations and congregational loyalty

People are at times reluctant to participate in interventions provided in a church they do not belong to. If this is the case then it is better to conduct the study on neutral ground [19].

#### Lack of trust

##### Work with multiple community organisations

- Distributed fliers at local churches, community events and speaking engagements [20].
- Our “sponsored” appearances through established gatekeeper community organizations and venues promoted trust in the study and the team [24].
- Physician colleagues approached a local organization; the Black Physicians Network and the local chapter of the National Association for the Advancement of Coloured People (NAACP) [20].
- The researchers built ties with Community Health Advisors who helped to educate and gain the trust of African-American communities [23].
- Based on advice from Tuskegee University Centre for Bioethics in Research and Health care, we organised discussions and focus groups with community leaders to develop guidelines for the ethical conduct of the study [23].

#### Stigma

##### Avoid using stigmatizing and embarrassing terms

All presentations and flyers should avoid using stigmatising terms like “mental illness”. Rather, more appropriate language can use terms such as “*deprimida*” (depressed), “*ataques de nervios*” (nervous or panic attacks), or “who had emotional troubles” for Puerto Rican and Mexican women [24].

##### Setting up an advisory board

An advisory board can help improve researchers’ understanding of cultural barriers and collaboratively develop strategies to overcome the fear and stigma often associated with research in minority communities [19].

#### Gender issues

##### Gender matched staff

Mothers were contacted by female research assistants who were warm, friendly, interested and made reference to the family situation to demonstrate empathy [16].

#### Psychopathology/substance misuse*

Addressing this barrier was beyond the scope of any of the research teams; this needs addressing at the level of health service provision.

#### Fear of being reported to immigration

##### Avoid direct questioning

We avoided asking questions about immigration status in the demographic questionnaire to alleviate any concerns [18].

### Practical issues

#### Lack of child care

##### Help with child care arrangements

- Free child care arrangements were made on site or at home [16].
- Offering participants reimbursement for their inconvenience (like a babysitting service) is helpful [23].

#### Transport provision

##### Help with transport arrangements

- Free transportation to and from intervention delivery sites was provided [16].
- Participants were offered free transportation vouchers [21].
- Reimbursements to cover for transportation costs were provided [22].
- Sessions were held at a local centre and close to transportation links [20].

#### Financial constraints

##### Financial reimbursement for time

Assure the participants that they would be compensated for time spent on assessments. Pay nominal costs to cover for transportation and time [22]. Eligible participants were paid $5 [17], $10 [20] for agreeing to participate.

##### Plan easily accessible location for meetings

On the recommendation of our community link, we organised a conference at an easily accessible location with minimal cost to participants [20].

##### Provide toll free phone contact

For every outreach effort we provided a toll-free phone number to call if participants wished to self-initiate contact with us [21].

#### Culturally inappropriate incentive

##### Make incentives culturally acceptable

Participants at times feel pressured by the extra obligation caused by money. To ease the awkwardness, try creating the perception of a decent gift or token of gratitude, rather than a payment.

- Put the money into a red envelope that is culturally used as a gift-wrap among Chinese people [22].
- Select incentives that are culturally appropriate like a small vinyl change purse, a magnetic refrigerator clip, a small fabric lunch bag, a T-shirt; all items can carry the study logo [24].
- All mothers and babies during the course of the study received birthday cards [16].

#### Medical insurance

##### Support people without medical insurance

- All interventions were provided free of charge [16].
- Interventions were marketed as free of charge services in locations throughout the region [21].

#### Lack of time

##### Beware of religious and social commitments

- When scheduling assessment interviews, try to anticipate likely religious holidays, vacations, visits to relatives and planned medical procedures, when participants may be unavailable [19].
- Each participant was requested in advance to inform us about holidays, celebrated or observed, so that we could recognise these special days [24].

#### Location of interview

##### Flexibility and choice of location

- Based on recommendations from our consumer council, the researchers were flexible around location of assessments [19].
- We assured participants that to better accommodate their schedules and concerns, interviews would be conducted at a location of their choosing [24].

#### Employment status

##### Flexibility around employment needs

The study team were flexible when arranging interview times by offering evenings or weekend appointments when participants could not attend during the week [23].

### Family/community related

#### Husbands’ influence

##### Work collaboratively with husbands

In case a husband disapproves of participation, discuss this issue directly with him after seeking due permission. Often the “man of the family” needs to be brought on board to facilitate recruitment [16].

#### Family perspectives

##### Engage and educate the family

- The Principal Investigator made an initial home visit to explain the study to the participants and their family. This helps to build a warm and trusting relationship [16].
- Providing clarification and information about study interventions to family members reduces the chances of participant refusal. Provision of incentives, reimbursements and cultural adaptations need to be highlighted [22].

#### Stigma for family

##### Building trust and confidence

- Establishing relationships with Latino agencies may inspire “*confianza*” (trust and familiarity) with the Latino community. This may facilitate the professional’s initial encounters with potential participants [21].
- Engaging with the community through multiple sources helps to overcome the stigma and mistrust barriers associated with research [19].

### Health service related

#### Underutilization of mental health services

##### Approach people outside the health services

- Our bilingual and bicultural staff attended several health fairs and ethnic community festivals. Flyers were distributed and announcements describing the research program were made in both Spanish and English [21].
- Researchers worked with Spanish specific media to reach out to a wider audience [21].

#### Language of professional/intervention

##### Providing language choice

- When aiming to recruit and deliver interventions in monolingual Spanish-speakers it is an essential pre requisite that all interventions are conducted in Spanish. Because of our bilingual and bicultural staff, retention rates were similar across all ethnicities [16].
- We employed researchers from different countries of origin, which facilitated working with participants from varied cultural backgrounds [18].
- It was deemed to be of critical importance whilst selecting staff that researchers were fluent in both English and Spanish [18].

#### Lack of communication and cultural awareness between staff and participants

##### Utilising researchers’ cultural communication skills

- We found that during our radio shows the anonymity of a dialogue between a patient and a clinician, or between a clinician and an audience was a very useful way to discuss sensitive health issues. A series of radio programs helped raise awareness of certain health issues in the community [22].
- Using predominantly bilingual and bicultural research and clinic staff at trial sites resulted in increased numbers of screening interviews [18].

#### Staff personal attributes

##### Developing skills to address misunderstandings

Often communities harbour misunderstandings and false allegations of exploitation. Field researchers should be trained and prepared to address these issues [23].

##### Demonstrating culturally appropriate interpersonal skills

- Research staff was trained to address older Latinos with respect, using formal titles, while being warm and personable. The polite form of the word “you” (“*usted*”, plus the related verb forms) were used to express respect, warmth and closeness. Other personalised touches like mentioning names of participant’s children can be extremely beneficial in fostering an engaging relationship [16].
- The Latino cultural value of “*personalismo*” (an emphasis on and an expectation of closeness in personal relationships) clearly facilitates consent to participate. This fosters collaborative relationships between researchers and participants that no other recruitment method like media advertisements or non-professional referrals can achieve [21].
- Latinos may find traditional formal approaches to be too informal and cold. They often positively respond to the “*simpatía*” of others. An individualized approach like telephoning participants periodically over the course of the study by a warm and friendly female research assistant was followed. During the conversation references were made to their family situation that conveyed a feeling of personal rapport [16].
- Researchers should provide a dignified, warm, and personalised approach (*personalismo* and *simpatía*) while communicating with participants [18].
- A number of times Latino women felt uncomfortable saying “no” and responded with a passive “yes” to appointments. Again this is a form of “*simpatía*” where one cannot say no because of wanting to preserve the interpersonal relationship and adopt socially desirable behaviour. Research staff should be trained to be culturally aware of this behaviour and expect no shows to the appointments [16].

### Research process

#### Limited willingness and enthusiasm of researchers

##### Collaborative working with recruiting clinicians

- The Principal Investigator was based for 0.5 day per week within the medical clinic with an aim of developing working relationships and organising teaching sessions on cross-cultural skills in both identifying depression and the consultation process [16].
- Develop working partnerships with existing services for older Latinos by providing training for clinical staff [21].

##### Understand the work process at the recruitment site

- Try to understand the existing workload of clinical staff at recruitment sites and ascertain the added burden due to working with ethnic minority participants [22].
- To save time and effort, try to integrate the screening protocol into the routine care pathways in the clinical sites [18].

#### Understanding the need for ethnic minority participation

##### Raising awareness about the importance of ethnic minority participation

- Research teams need to emphasise the unique impact of involving ethnic minorities in the study by highlighting the suspected risks and compelling clinical needs. Culturally sensitive aspects of the study and the expected benefits should be emphasized. This can be presented in the form of a pamphlet to reinforce the verbal presentation [22].
- Avoid overemphasizing the academic needs of research as this could be misinterpreted as a way of serving researcher goals and not true needs [22].

#### Paucity of resources available

No direct solution to this issue was made in the included papers.

#### Appropriateness of assessment tools

##### Cultural appropriateness of assessment tools

- Instrument selection is crucial with African-American participants with very little (if any) experience of clinical research participation. We initially undertook pretesting with highly structured interviews. There was wide variation in interview length and respondent burden. Thus we gave up on their use and took a more flexible approach to the diagnostic assessment [17].
- A standardized screening procedure was undertaken by bilingual/bicultural recruiting staff, in the participant’s language of choice [21].

#### Non-availability of translated materials

##### Developing newly translated research instruments

- Using back and forward translation techniques, research instruments were carefully translated into Spanish [16].
- Initially we selected familiar Chinese replacement words for the English ones. Their use made the translations culturally appropriate to match the respondent’s linguistic needs [22].
- Our advisory board provided guidance on the development of appropriate study instruments and translations. All cards and other materials were personalized and offered to participants depending upon their language of choice [24].

##### Using previously translated research instruments

d) We have previously developed a consensus and psychometrically tested translations by a team of experts. We have used a cross-section of Latino culture experts to ensure appropriate Spanish translations for participants originating from different Spanish-speaking countries [16].

#### Lack of culturally competent staff

##### Delivering cultural competency training to researchers

- We provided high quality, culturally sensitive training to the research interviewers. The training covered topics such as how to approach patients, how to introduce the study and the most appropriate way to communicate about confidentiality and voluntary participation. The research interviewers should be well trained in research skills and experienced in working with ethnic minority participants [17].
- Researchers with varied educational backgrounds were trained on how to conduct research interviews with older ethnic minority participants. In our experience trained researchers performed on par with ethnically matched recruiters [19].
- Matching researchers did not influence interview completion rates, however this may be more important for intervention delivery. Researcher selection and training can compensate for not being able to ethnically match researchers [21].
- Well trained staff should be culturally sensitive (using *personalismo*) particularly when approaching women [18].
- Employing staff familiar with the ethnic minority community and later training them around immigration and cultural sensitivity helps to improved recruitment [24].

#### Lack of culturally matched staff

##### Employing experienced staff

- We employed researchers with past clinical experience of working with African-Americans, as this minimized the chances of any culture related problems arising during the study [17].
- The team should employ experienced, ethnically matched researchers as potential participants feel at ease sharing personal information with someone from their own background. Ethnically matched researchers tend to be more sensitive to participants’ needs and can often act as advisors to the senior investigators on how to devise recruitment and retention strategies [19].
- Researchers with extensive experience of working with the Latino communities, with ethnic minority social and political organizations, who were also aware of social hierarchies, were employed. This knowledge enhanced their effectiveness as recruiters [24].

#### Under-representation of ethnic minorities at recruitment sites

##### Site selection

- We used population projection data based on the national census to calculate absolute numbers of African-Americans per state/county in the selected regions. Geo-coding and census data were linked to digital maps to visualize the ethnic minority population. This enabled us to select recruitment sites mapped to population densities and estimate a recruitment radius for each site, avoiding overlap with an adjacent site [23].
- Medical settings situated in and serving a district, with a large Latino population were specifically selected [16]. Our researchers recruited an urban clinical site with a high ethnic minority density, resulting in successful recruitment rates of African-Americans [17]. Similarly, recruitment was carried out from a prenatal care clinic that comprised of 60% Latinas [18], as well as from academic medical centres that were located in regions with predominantly large and research naive African-American population [23].

##### Liaise with local clinical staff

- Clinicians and counsellors serving mentally ill ethnic minority women were informed about the study and requested to facilitate recruitment [24].
- Links were established with recent graduates of the residency programs and with ethnic minority professional organizations (Association of Black Psychiatrists and the Michigan Psychiatric Society). Professional referrals resulted in higher recruitment, as compared with media efforts and non-professional referrals [17].
- Enthusiastic clinicians who agreed to provide a treatment site proved to be better recruiters [22].

##### Plan wider interaction with local sites and organisations

- Before commencement of recruitment at clinical sites, investigators organised multiple meetings with clinical staff and leaders to understand the inner workings at each site, assessing feasibility and barriers to ethnic minority recruitment [18].
- To compensate for expected low numbers of possible ethnic minority participants, we planned a wider engagement strategy. Engagement presentations were delivered in diverse settings, such as language classes, vocational classes, churches and support groups. Printed flyers were also widely distributed in churches, nightclubs, government assistance offices, social service organizations, laundries, social clubs, beauty salons, restaurants, grocery stores and other locales [24].

#### Understanding of the consent process

##### Train staff in consent procedures

- Recruiting field researchers were extensively trained on procedures for obtaining written consent [17].

##### Adopting a culturally sensitive approach

- We ensured that the participants fully understood the intention and content of research consent forms. Linguistically alien and culturally threatening wordings were replaced in the consent forms. Researchers spent a lot of time verbally explaining the purpose of obtaining written consent and removed fears about research participation [18].
- Consent forms were translated into Chinese and we found that it is often helpful to have a family member in attendance when obtaining consent [22].
- As a result of feedback received from the pilot testing of consent forms, we changed the word “*investigación*” to “*un studio*” (study) to reduce the stigma associated with participating in research [18].
- To satisfy participant’s fears about confidentiality and explicit consent, after approval from our funding source we included a Certificate of Confidentiality in our consent pack [18].

## Discussion

Our previously published systematic review on barriers to ethnic minority recruitment in mental health provided us with an opportunity to look in depth into the interplay of various barriers to recruitment. It became evident that there is significant overlap between categories and they are often interlinked. This may suggest that multi-component strategies are needed to overcome these barriers.

Having developed a broad classification of barriers in the initial review, we next revisited the original papers to extract data on proposed solutions to these barriers. We made an attempt to find solutions related to each barrier and (as mentioned earlier), we found that solutions to these barriers were again overlapping, interlinked and often multifaceted. It is possible that, as the barriers are overlapping, that a strategy to overcome one barrier can in fact make a positive impact on others. However, it would be a mistake to assume that only a few recruitment strategies are needed to increase recruitment rates.

As a specific example of this, the introduction of culturally sensitive methods at the time of recruitment, without matching cultural adaptations in the intervention itself may reduce the impact achieved. Participants may consent to join the study, but if they find the intervention clashing with their cultural norms, their chances of continuing in the study would diminish.

Based on our own research experience and findings of these two reviews, it is important that researchers plan strategies to overcome these barriers at the proposal writing stage. Once the project has begun, providing extra human resources and meeting additional costs may be beyond the financial and staffing capabilities of the project. Initiating culturally sensitive strategies at the start of the study is not enough; the team should regularly monitor recruitment rates and if possible quantify the cost effectiveness of each strategy on enrolment into the study. This will help in identifying ineffective strategies and help direct funds into strategies that are effective [25,26].

In examining the barriers and the proposed solutions, it becomes evident that the solutions can be categorised based upon human and financial costs. We would like to propose the following:-

- Solutions that are not culturally specific and may also apply to majority ethnic (i.e. ‘white’) participants: This means that the proposed solutions are basically adopting good practice and apply to the general population but a cultural emphasis is needed when recruiting ethnic minority participants. Strategies like community engagement, provision of child care, transport and incentives will fall under this category.
- Solutions requiring additional resources specifically for ethnic minorities: Various types of solutions, such as procurement of extra materials, translations, adaptations and provision of multilingual staff will need extra financial input.

What we have documented in this paper is the accounts of authors about how they devised various strategies to improve recruitment. Authors have generally not been able to quantify the positive impact of these strategies on recruitment in a rigorous way that would demonstrate clearly their advantages over routine methods. There have been a number of published trials conducted to evaluate recruitment strategies [27], but none have reported the effects of interventions for ethnic minority participants. A key next step to further the science of recruitment in this area is the formal testing of these strategies, using appropriate experimental or quasi-experimental methods. The optimal way of doing this is to conduct nested trials of recruitment interventions in ongoing randomised clinical trials, where some patients in the trial are randomly assigned to an ethnically sensitive method of recruitment, and some receive a standard model. In this way, the effectiveness and cost effectiveness of the various strategies may be formally tested [28]. There may be some barriers to the use of such nested trials – for example, ethical concerns may arise if culturally appropriate recruitment is to be randomised. Other forms of evaluation may be required at times [29]. However, demonstrating the impact of these strategies will be important in encouraging their uptake among research teams and funding agencies, especially where the strategies are associated with significant costs.

Another important point worth considering is that we need to further increase cultural sensitivity of research in ethnic minorities outside the US. It is a fact that ethnic minority groups are found in almost all developed countries, but their countries of origin vary considerably: in the US we mainly come across people of African-Caribbean and Spanish descent; in the UK the majority are South Asians; while in Europe there is a large representation from North Africa. There will be some commonalities in barriers to recruitment and strategies to overcome them, but there are clear cultural differences between these groups. In addition, there is a wide variation in health systems and a number of barriers are related to the peculiarities of the health service, highlighting the need for solutions to be tailored according to the structure of the health service under study.

### Limitations

The main limitation of this review is that we have only given verbatim description of various strategies to overcome ethnic recruitment barriers as described by the original authors. Quantitative impact of these strategies on recruitment rates and the extra costs to implement these strategies were not fully described by the authors.

## Conclusions

It can be a challenge to engage ethnic minorities in research; challenges that may be confounded in the context of mental health issues. Investigators need to employ a range of strategies to overcome these barriers, and our proposed typology of strategies provides some guidance. The strategies in this paper may not just be relevant for mental health patients alone and further evaluation of their impact in research across disciplines and in lower and middle income countries is also required.
